# iPSCs-Based Neural 3D Systems: A Multidimensional Approach for Disease Modeling and Drug Discovery

**DOI:** 10.3390/cells8111438

**Published:** 2019-11-14

**Authors:** Gianluca Costamagna, Luca Andreoli, Stefania Corti, Irene Faravelli

**Affiliations:** Dino Ferrari Centre, Neuroscience Section, Department of Pathophysiology and Transplantation (DEPT), University of Milan, Neurology Unit, IRCCS Foundation Ca’ Granda Ospedale Maggiore Policlinico, 20122 Milan, Italy; gianluca.costamagna@unimi.it (G.C.); luca.andreoli95@gmail.com (L.A.); stefania.corti@unimi.it (S.C.)

**Keywords:** brain organoids, neurological disorders, iPSCs, drug discovery, disease modeling, neural chimera

## Abstract

Induced pluripotent stem cells (iPSCs)-based two-dimensional (2D) protocols have offered invaluable insights into the pathophysiology of neurological diseases. However, these systems are unable to reproduce complex cytoarchitectural features, cell-cell and tissue-tissue interactions like their in vivo counterpart. Three-dimensional (3D)-based culture protocols, though in their infancy, have offered new insights into modeling human diseases. Human neural organoids try to recapitulate the cellular diversity of complex tissues and can be generated from iPSCs to model the pathophysiology of a wide spectrum of pathologies. The engraftment of iPSCs into mice models and the improvement of differentiation protocols towards 3D cultures has enabled the generation of more complex multicellular systems. Consequently, models of neuropsychiatric disorders, infectious diseases, brain cancer and cerebral hypoxic injury can now be investigated from new perspectives. In this review, we consider the advancements made in modeling neuropsychiatric and neurological diseases with iPSC-derived organoids and their potential use to develop new drugs.

## 1. Introduction

Recent technological advances achieved in stem cell research have provided unprecedented means to study the nervous system, both in vitro and in vivo. The enthusiasm for stem cell-based technologies rose with the development of embryonic stem cells (ESCs) cultures, followed by human-induced pluripotent stem cells (iPSCs) and, lately, by ESCs- and iPSCs-derived three-dimensional (3D) culture systems.

Human ESC lines were first isolated in 1998 [1] and differentiation protocols towards multiple tissues were soon designed, aiming to eventually develop allogeneic cell-based therapies to several degenerative diseases. As for neural disease modeling, ESCs were successfully differentiated to neural precursors [2] and many neuronal subtypes, e.g., dopaminergic neurons [3] and motor neurons [4], as well as astrocytes [5], oligodendrocytes [6] and microglia [7]. However, ESCs advantages were offset by the need of genetic manipulation to introduce disease-relevant mutations and their limited supply [8].

Human iPSCs reprogrammed from patients’ somatic cells such as fibroblasts and blood cells [9,10,11] have given new stimuli in many fields of neurobiology: they provided researchers with patient-derived human stem cells offering a more scalable supply for culturing systems and the theoretical possibility of personalized autologous therapies for a wide spectrum of diseases [12]. Moreover, iPSCs can be differentiated into cells able to recapitulate the hallmarks of pathological cells and tissues to develop disease models and test new potential therapies [13]. Many neural diseases have already been modeled using iPSCs and their pathological features thoroughly described: hyperexcitability, altered axonal transport and increased apoptosis in spinal muscular atrophy (SMA) neurons [14,15]; elevated lysosomal activity and higher response to glutamate in iPSC-derived neurons from Huntington disease patients [16,17,18]; decreased dendritic length and altered calcium signaling in neurons derived from patients with Timothy syndrome (TS) [19]; altered mitochondrial activity, abnormal mRNA expression and lithium-responsive hyperexcitability from patients diagnosed with bipolar disorder [20,21]. These phenotypes are reproducible, scalable and disease-relevant, offering an important insight into some intrinsic pathological mechanisms at a cellular level.

**Figure 1:**
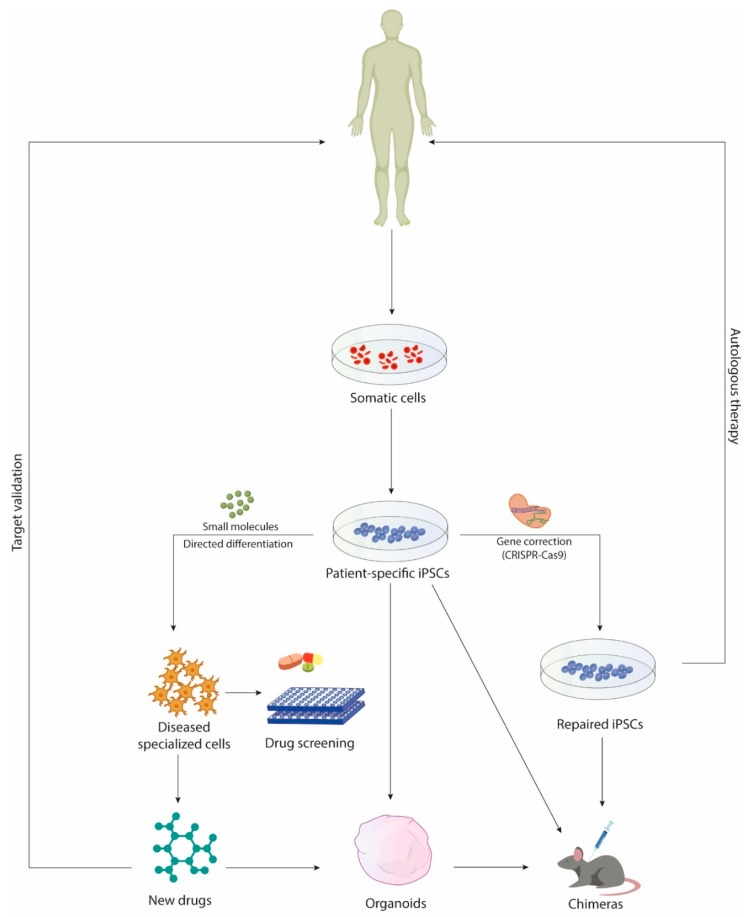
Drug discovery based on induced pluripotent stem cells (iPSCs) and iPSC-derived systems. The improvements made in iPSCs culturing and differentiation methods have increased the efficiency and the quality of iPSCs lines. In addition, the use of gene correction technologies such as CRISPR-Cas9 and specific small molecules has enabled the generation of patient isogenic lines and terminally differentiated cells, reducing background genetic variability and broadening the spectrum of cells available for drug screening. Currently, some candidate therapies discovered with iPSC-based platforms and chimeric models are being tested in human clinical trials. In parallel, optimization in the efficiency and scalability of clinical-grade cells has led to iPSC-derived neural cells transplantation in humans.

Although these systems have increased the understanding of different diseases, human pathologies arise in the context of complex interactions at a cell-, tissue-, organ- and host-pathogen level. Therefore, new culture systems are being developed to more closely recapitulate dysfunctions at organ- and tissue-level, enabling new approaches to disease modeling and compound screening (Figure 1). Recently, 3D culture methods have been implemented, primarily leading to the generation of organoids [22,23,24], a complex self-organizing 3D aggregate of different cell types derived from ESCs or iPSCs capable of going through the differentiation and morphogenesis pathways down to recapitulate core features of full-grown tissues. The first in vitro attempt to grow 3D neural tissue dates back to 2008, when the method of serum-free floating culture of embryoid body-like aggregates with quick reaggregation (SFEBq) was tuned [25]. In 2013, Lancaster et al. discovered that embryoid bodies embedded in Matrigel, in absence of small molecules prompting specific regional patterning, gave rise to neuroepithelial buds subsequently maturing in different brain regions [22]. In recent years, new data have been provided regarding organoid generation and patterning [22,26,27]. Indeed, several groups have developed multiple differentiation protocols to generate varying central nervous system (CNS) regions including ventral forebrain [28], midbrain [29], hippocampus [30], hypothalamus [29], dorsal cortex [31] and spinal cord [32].

In addition, different neural organoid-based researches have tried to model neurological diseases and neurodegeneration [33]. Although it is unclear how much insight can be gained from neural organoids to model neurodegenerative diseases, some studies suggest that they may be relevant in recapitulating some late-onset phenotypes, such as Alzheimer disease (AD). The main neuropathological features of AD are neurofibrillary tangles composed of hyperphosphorylated tau protein and the extracellular accumulation of amyloid-β peptides [34]. 2D cultures may not be suitable to model the complex extracellular compartment required to reproduce the extracellular amyloid deposition, making 3D cultures potentially promising alternatives. For example, a research investigating familial AD (fAD) mutations of β-amyloid precursor protein and presenilin 1 has shown extracellular deposition of amyloid-β in a human stem cell-based 3D culture system [35]. Moreover, neural cells differentiated within this 3D culture system present both filamentous and phosphorylated tau protein aggregates. Consistently with these findings, also neural organoids from murine iPSCs display high levels of ptau and extracellular β-amyloid [36]. Raja et al. has obtained similar results from fAD patient-derived neural organoids, exhibiting AD-like phenotype such as hyperphosphorylated tau aggregation, endosome abnormalities and extracellular amyloid accumulation [37]. These results are important because they are difficult to reproduce in mice models of fAD [33,38] and suggest that neural organoids are a suitable model to recapitulate some aspects of fAD-related phenotype.

In parallel to disease modeling, the development of xenotransplantation, that is the transplantation of cells from a species to different species, is expanding the possibility of generating even more complex iPSC-derived biological systems, the so-called chimaeras. Chimeric models allow one to study iPSCs-derived cells and organoids integrated within the CNS in a more physiological environment, where they are perfused by the host vasculature and interact with microglia and surrounding neural networks [39,40]. Consequently, engrafted organoids could more faithfully recapitulate differentiation as a result of the exposure to morphogenetic cues and to sensory signals conveyed through the host neural system.

In this review, we consider the advancements made in modeling neuropsychiatric and neurological diseases with iPSC-derived organoids and their potential use to develop new drugs. The engraftment of iPSCs into mice models and the improvement of differentiation protocols towards 3D cultures has enabled the generation of more complex multicellular systems. As a consequence, models of neuropsychiatric disorders, infectious diseases, brain cancer and cerebral hypoxic injury can now be investigated from new perspectives (Table 1).

## 2. Modeling Neuropsychiatric and Neurodevelopmental Disorders

Despite progress in neuroscience, the biological basis of neuropsychiatric disorders is still elusive [51]. Two of the main challenges to be faced are the complexity of the human brain, containing different types of specialized cells and connections, and the heterogeneous factors playing a causative role in neuropsychiatric diseases. The latter includes an intricate interplay of environmental, genetic and psychosocial factors, difficult to reproduce in animal models or in vitro [52]. The lack of neurobiological markers represents another limitation, since many disorders are labeled into clinical categories by behaviors and self-reported disturbances [53]. Moreover, neuropsychiatric disorders present obstacles both in treatment and drug development in reliable models [54].

Recent techniques offer the opportunity to study nervous system diseases from new viewpoints. For example, genomic analysis has contributed to the definition of copy number and genetic variants as risk factors for increased susceptibility to neuropsychiatric conditions [55]. In addition, clustered regularly interspersed short palindromic repeats (CRISPR)-Cas9, a revolutionary genome engineering tool, has led to efficient and precise genome changes for the study of gene-function correlations both in vitro and in animal models [56]. In parallel, iPSCs can be derived from selected patients with increased disease susceptibility by carrying accumulation of common variants with small effect or rare ones of large effect, such as in schizophrenia (SCZ). Different studies on 2D and 3D cultures have provided important advancements in modelling SCZ [57]. For example, iPSC-derived forebrain organoids from SCZ patients show disrupted neocorticogenesis [58]. Particularly, they exhibit (i) enhanced proliferation of neural precursor cells (NPCs), (ii) reduced expression of reelin, a protein regulating migration towards the cortex, (iii) decreased cortical neuron development in favor of a subcortical pattern and (iv) morphologically altered interneurons, which support the connection between cortical columns. These findings are consistent with 2D-based studies showing impaired migration, proliferation and differentiation of iPSC-derived NPCs and neurons from SCZ patients [57].

Although environmental influences remain to be elucidated, examples that 3D-culture protocols can be integrated with gene editing technologies and refined to model specific aspects of neuropsychiatric diseases include Timothy syndrome, autism spectrum disorders (ASDs) and tuberous sclerosis complex (TSC)

### 2.1. Timothy Syndrome

Brain organoids can model monogenic causes of ASD enabling the investigation of gene-phenotype relationship during human cortical maturation.

Recently, Birey et al. have modelled Timothy syndrome, a genetic disease caused by a mutation in the l-type calcium channel CACNA1C associated with epilepsy and ASD, using iPSCs-derived ventral and dorsal forebrain organoids [41]., Cortical maturation involves the specification and functional interplay between glutamatergic neurons in dorsal forebrain (pallium) and gamma-hydroxybutyrate (GABA)-releasing interneurons in ventral forebrain (subpallium) [59]. The differentiation towards a ventral or dorsal forebrain fate has been assessed using immunostaining and single-cell transcriptional profiling. Particularly, dorsal forebrain organoids show increased expression of intermediate progenitors (TBR2 and HES6), dorsal progenitors (LHX2, PAX6, GLAST1) as well as glutamatergic neurons (VGLUT1^+^) exhibiting cortical layer markers (TBR1 and CTIP2). Instead, ventral forebrain organoids present typical subpallial markers (GABA, GAD67, somatostatin, calretinin and calbindin), known markers identifying GABAergic interneurons [41].

**Figure 2:**
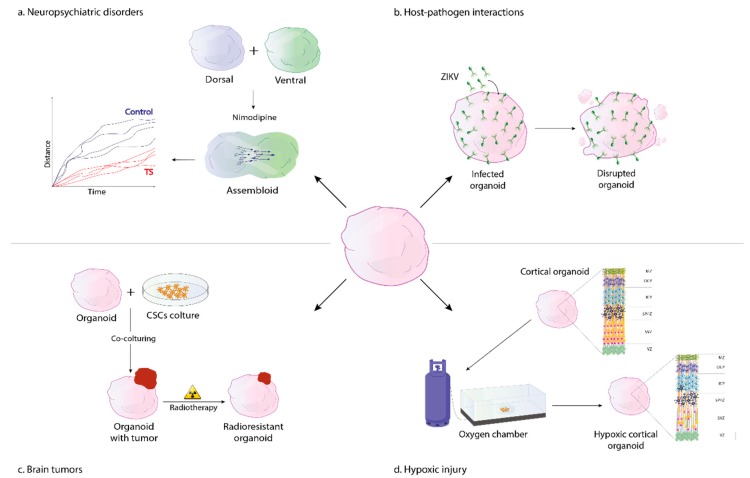
iPSC-derived neural organoids in disease modeling. The figure shows examples of different diseases modelled using neural organoids. Neuropsychiatric disorders, such as Timothy syndrome, can be modelled with dorsal and ventral forebrain organoids generating assembloids, which exhibit alterations in neuronal saltatory migration that can be corrected with the l-type channel blocker nimodipine (upper left corner). Zika virus-host interaction in human neural organoids leads to morphological and cytoarchitectural abnormalities (upper right corner). Neural organoids can be co-cultured with cancer stem cells (CSCs) to model human brain tumors and test therapeutic approaches such as radiotherapy (lower left corner). Cortical organoids exposed to low oxygen concentration in gas chambers recapitulate some cytoarchitectural abnormalities present in fetal hypoxic injury (lower right corner).

After differentiation, interneurons migrate during fetal development from the ganglionic eminence to the developing neocortex undergoing maturation and integration into cortical circuits [60]; early circuit alterations can lead to neuropsychiatric manifestations including ASD and epilepsy. Assembling ventral and dorsal forebrain organoids into so called “assembloids” enables to model the integration of GABA interneurons into functional microcircuits and their saltatory migration into the cerebral cortex during brain development [41,61]. Saltatory migration is a cyclical movement of an extension of the neuronal leading process in one direction followed by a transient swelling of the soma and nuclear translocation (nucleokinesis) [62]. These steps are repeated generating the typical saltatory pattern of migrating neurons. When assembled with dorsal forebrain organoids, TS ventral forebrain organoids labeled with a Dlxi1/2b-GFP^+^ reporter of GABA interneurons show a disrupted saltatory pattern, culminating in delayed neuronal migration on live imaging. Blocking l-type calcium channel with nimodipine or roscovitine restores the phenotypes in diseased assembloids [41] (Figure 2).

In the same study, Birey et al. have also investigated the functional properties of TS assembloids using calcium imaging and whole-cell patch clamping. Similarly to results in hiPSC-derived TS neural cells [19], neurons from TS assembloids exhibit increased residual calcium in response to depolarization. Moreover, TS assembloids present functional synapsis, showing integration of ventral and forebrain organoids into neural networks responsive to electrical stimuli. These results are important because indicate the possibility of generating active and synaptically connected human microcircuits in 3D cultures.

This research shows how neural organoids can be differentiated into more specialized brain regions modeling specific neurobiological processes. Particularly, by modifying culture conditions of human organoids, several protocols have been used to direct differentiation towards the cerebral cortex [35], the ventral and dorsal forebrain [41], the cerebellum [63] the midbrain [64] and the basal ganglia [65]. These directed brain organoids can be assembled in multicellular structures reproducing cell-cell interactions and neural circuitry that can be useful to model diseases where cell migration and neural circuitry disruption seem to play an important role such as in neurodevelopmental disorders.

### 2.2. Autism Spectrum Disorders

Autism spectrum disorders (ASD) refer to a group of neurodevelopmental disturbances defined by difficulty with communication with other people, restriction in interests and repetitive behaviors, affecting the patient’s ability to function properly in different areas of life. Genetic hereditability is a risk factor for ASD and several copy number variants and different mutations have been associated with subtypes of ASDs with varying penetrance and variable expressivity [49,61].

Almost 90% of all ASD cases are considered idiopathic and few studies have tried to model them [66]. In a study by Mariani et al., iPSC-derived dorsal telencephalic organoids have been generated from ASD patients with macrocephaly and no ASD-related mutation as shown by whole genome analysis [36]. ASD organoids have been characterized using immunochemistry, electrophysiological and transcriptomic analysis. Transcriptomes of ASD organoids have been compared both with controls and BrainSpan, a dataset of postmortem human brain transcriptomes from embryonic age to adulthood [67]. ASD organoids show dysregulated transcripts implicated in cell proliferation, neuronal differentiation, synaptic transmission and reflect the transcriptome of the human dorsal telencephalon (cerebral cortex and hippocampus) during early fetal development (9 weeks post-conception) [43]. Moreover, immunohistochemistry techniques reveal an increased number of progenitors, GABAergic neurons and a transient increase in size during maturation. In parallel, as shown using whole-cell patch-clamp recordings, ASD neural organoids present functional synaptic connections. Particularly, most neurons in the organoid fire only a single action potential, but some show multiple spikes and spontaneous synaptic currents. In addition, they present an enhanced expression of GABAergic phenotypes electrophysiologically, consistent with an increased presence of a specific sodium channel isoform in ASD organoids [43]. Differential gene expression analysis on ASD organoids shows that one of the most upregulated genes is FOXG1, an important regulator of forebrain differentiation linked to ASD-like neurodevelopmental syndromes [62,63]. The attenuation of FOXG1 expression by short hairpin RNA reverts the abnormal high presence of GABAergic neurons [36]. This study highlights how human iPSC-derived cortical organoids recapitulating first trimester brain development can be useful in assessing the formation of functional synaptic connectivity as well as the altered dynamics of brain growth in idiopathic ASD presenting with macrocephaly.

In another study, telencephalic organoids derived from iPSCs carrying a CRISPR-cas9-induced heterozygote mutation of CDH8 (an ASD-related chromatin remodeling factor) have been used to model a form of non-idiopathic ASD [68]. Derived organoids exhibit increased expression of genes involved in differentiation of GABAergic neurons, consistent with the results obtained in idiopathic ASD [43], suggesting the presence of common molecular pathways in different clinical conditions caused by apparently unrelated genetic background.

These observations illustrate how 3D cultures coupled with gene editing techniques may be exploited to investigate the molecular basis of genetically heterogeneous disorders such as ASDs.

### 2.3. Tuberous Sclerosis Complex

Tuberous sclerosis complex is an autosomal dominant, systemic disease characterized by epilepsy, ASD, delayed intellectual development and psychiatric manifestations associated with mutations in TSC1 and TSC2 genes [69]. TSC1 and TSC2 mutations lead to an increased activation of mammalian target of rapamycin (mTOR) complex, a serine/threonine kinase involved in cell proliferation and metabolism in response to growth factors and nutrients [70]. Cortical tubers, which are focal areas of disorganized and dysplastic neurons, glia and giant cells in cortical layers, are the hallmark of TSC [71].

Recently, cortical organoids have been generated to model tuberous sclerosis by introducing TSC1 and TSC2 mutations with CRISPR-cas9 editing in hESCs and from patient-derived iPSCs [72]. Organoids carrying heterozygous mutations of TSC1 and TSC2 show no abnormalities in neuronal or glial differentiation, while homozygous knockout organoids display an altered balance of neurons and glia with reduced expression of neuronal markers and hypertrophy of glial lineages. Both neurons and glial cells within brain organoids are highly dysmorphic and increased in size over time, similarly to observations in cortical tubers from patients’ samples [71]. Organoids obtained from patient-derived iPSCs with TSC2 heterozygous mutation and edited with a mutated TSC2 conditional allele prove that biallelic inactivation of TSC2 is needed to reproduce the phenotype at a cellular level [72]. These results are in line with loss of TSC1 and TSC2 heterozygosity leading to dysplastic cell formation in cortical tubers [73]. Rapamycin and its derivative everolimus (also called rapalogues) can be used to treat both epilepsy and subependymal giant cell astrocytomas, glioneural brain tumors occurring in 20% of TSC patients [74]. However, if the treatment is stopped the tumor can regrowth [73]. Rapamycin in TSC organoids prevents mTOR hyperactivation in early-stage TSC2 homozygous knockout cultures and rescue cellular hypertrophy, but later treatment does not, suggesting a critical therapeutic window for mTOR to regulate cell differentiation in brain development. In addition, removal of rapamycin in early-treated organoids is associated with mTOR hyperactivity, suggesting the potential need for chronic use of rapalogues to treat TSC [72]. Thus, neural organoids permit to investigate the effects of currently available drugs on a multicellular scale and can faithfully recapitulate cytoarchitectural features and genetic expressivity of complex neuropsychiatric disorders such as TSC.

## 3. Organoid Models of Neural Hypoxic Injury

The technological advances in neonatal care have increased the survival rates of extremely premature infants, defined as born before post-conception week 28. These critical developmental stages coincide with the formation of the human cerebral cortex and can be disrupted by hypoxic injuries. Hypoxic-derived encephalopathy (HE) presents with gray- and white-matter abnormalities correlating both with the cognitive outcome and behavioral disorders. Perinatal hypoxia, defined as a decreased partial pressure of oxygen (PO_2_) below 40 mmHg, is considered to be the main cause of HE, inducing molecular changes on susceptible cortical cells that still remain unknown [75]. Some challenges in solving this issue are related to the difficulty in directly investigating the pre-birth human brain and recapitulating its maturation in animal models.

In recent research, brain-region-specific organoids mimicking the developing human brain have been differentiated to human cortical spheroids transcriptionally resembling the midgestation brain [47] to model the effect of oxygen reduction on corticogenesis in premature newborns [47]. Specific organoid subregions such as the subventricular zone (SVZ), a highly proliferative area adjacent to the ventricular zone, where neurogenesis progresses until late gestation phase, are more susceptible to damage induced by oxygen deprivation. Particularly, low level of PO_2_ affect a specific neuronal subpopulation, TBR2+ intermediate progenitors, active cells residing the SVZ that are involved in increasing the number of cells in the neocortex (Figure 2). Interestingly, PAX6+ radial glia cells residing the ventricular zone, an organoid subregion organized around a lumen, are not susceptible to low oxygen exposure [47]. Another key aspect in this research is related to the role of the unfolded protein response (UPR) pathway in modulating cellular response to hypoxia. During phases of cellular stress such as in case of oxygen deprivation, the UPR pathway aims to restore protein homeostasis [68,76]. TBR2+ intermediate progenitors, which reside in a hypoxic environment, show an impaired activation of UPR, particularly of the PERK–eIF2α–ATF4 pathway, and present an early neuronal differentiation. Human cortical spheroids exposed to a small molecule known as integrated stress response inhibitor (ISRIB), which restores protein translation in case of low PO_2_, present a higher density of TBR2+ cells and limit the premature differentiation of the TBR2+ progenitors [47]. These results suggest that modulators of the UPR pathways such as ISRIB might be useful to improve hypoxia-related defects in specific cortical TBR2+ cell subpopulations. Moreover, although it remains to be determined how changes in specific progenitors affect brain development, this study suggests that cortical organoids could be used as models to evaluate cell susceptibility to hypoxia in fetal brains. Thus, 3D brain organoids can give valuable insights into investigating new potential therapeutics and the effect of environmental factors on brain development.

## 4. 3D Models of Host-Pathogen Interactions

Cells terminally differentiated from iPSCs are susceptible to infections with human pathogens, offering opportunities to investigate host-pathogen interactions. Human iPSCs models overcome the limitations of species-specificity of infectious pathogens and inflammatory responses, with a resulting translational potential [77,78]. IPSCs-derived neural organoids model virus-host interaction in the context of Zika virus (ZIKV) and Herpesviruses

### 4.1. Zika Virus

ZIKV has been linked to serious neurological diseases, including Guillain-Barre syndrome and to congenital malformations, such as microcephaly [79]. Despite clinical evidence, there had been no direct experimental proof showing that ZIKV is able to cause early brain defects until 2016 [80]. Since then, several studies have shown the importance of 3D cultures in elucidating the mechanisms of ZIKV infection. Three landmark publications in the field illustrate that ZIKV disrupts the generation of neurospheres, induces neural precursor cell death and reduces the overall growth of organoids [29,81,82] (Figure 2). Particularly, ZIKV exhibits a specific trophism towards SOX2+ neural precursor cells and induces a decrease in neuronal cell-layer volume, resembling microcephaly [29]. Recently, ZIKV has been shown to cause microcephaly as well as a lissencephaly-like phenotype in a human 3D model of cortical folding [83].

IPSCs-derived brain organoids have helped to unravel some of the underlying mechanisms of virus-induced microcephaly in humans. Impaired recruitment of centrosome proteins has been reported as a key mechanism in genetic induced microcephaly [84,85]. Similarly, ZIKV perturbs centrosome function, promoting incorrect orientation of the mitotic plane, leading to neural progenitor cells depletion as reported in a study using human neurospheres [86]. AXL protein is considered an important virus entry-receptor in NPCs as shown both in 2D- and 3D-based studies [21,87]. However, ZIKV infection in cerebral organoids is not affected by AXL ablation [88]. Accordingly, early treatment of forebrain organoids with inhibitors of AXL such as small molecules (R428) or blocking antibodies leads to limited effects on virus-induced disruption in neurogenesis [63].

Human neural organoids have proved to model ZIKV replication and disruption of neurogenesis underlying virus-mediated microcephaly, thus they are being used for testing potential drugs. Several compounds have been screened in 2D NPCs cultures, and emricasan, a pan-caspase inhibitor, has been the most effective suppressor of ZIKV-mediated caspase activity in vitro [89]. Though not inhibiting ZIKV replication, emricasan neuroprotective effect has been confirmed when tested on brain organoids [89]. In a different study, hiPSCs differentiated into NPCs have been exposed to ZIKV and screened for potential drugs blocking ZIKV infection [90]. Hippeastrine hydrobromide (HH) and amodiaquine dihydrochloride dihydrate present the highest efficacy. Forebrain organoids have been used to validate the ability of these two selected drug candidates with anti-ZIKV activity to rescue microcephaly-related defects [90]. Particularly, HH induces a decrease of progenitor proliferation, ZIKV-induced apoptosis and suppresses ZIKV copies to undetectable levels. In addition, ZIKV activates anti-viral immunity by triggering the production of small interfering RNA in hNPCs [91]. Accordingly, enoxacin, a broad-spectrum antibiotic working as an RNA interfering enhancer, exerts a strong anti-ZIKV effect, preventing infection and microcephaly-like phenotype in human brain organoids [91].

### 4.2. Herpesviruses

Herpesviridae family is a heterogeneous group of viruses with a tropism for critical organs such as hematological and vascular system, gastrointestinal tract and the nervous system and may cause severe complications in apparently healthy individuals. The outcome of herpesvirus infection can be dramatic when they reach some anatomical regions such as the CNS or in immunocompromised subjects, aged patients and newborns. Herpes simplex virus (HSV) and Cytomegalovirus (CMV) are two important herpesviruses leading to congenital infections and intrauterine growth restriction. Symptomatic infants at birth show different neurological disorders including mental retardation, vision loss, hearing impairment and microcephaly [92]. In addition, herpesvirus infections in neonates are associated with encephalitis and high mortality, despite early antiviral treatment [93]. Precise reasons for the increased severity of disease in newborns remain obscure [94]. Particularly, a poorly understood process in herpesvirus infection is the role of cell susceptibility and viral latency, involving close interactions between the virus and its host cell [95].

Cerebral organoids derived from iPSCs have been used to model CMV and HSV infections in vitro. Organoid cultures derived from CMV-infected iPSCs include the viral genomes and show decreased cellularity, cysts, vacuoles and areas of necrosis. Β-tubulin III, a post-mitotic marker of neural differentiation localized in the axon, presents an aberrant expression associated with a disruption of neural projections and lamination within the cortical layers of the organoids [40]. These results parallel observations in infected human clinical samples, reporting delayed neural maturation and abnormal cortical lamination [96,97], proving the valuable role of 3D cultures in modeling some aspects of CMV-induced pathology in developmental brain. Similarly, brain organoids have been used to evaluate HSV-1 latency and reactivation in vitro. 3D cultures can be efficiently infected with HSV-1, showing the susceptibility of MAP2-positive neurons to the lytic phase of infection [98]. Interestingly, the efficient chemically induced reactivation of HSV-1 latent phase in 2D cultures is limited in 3D systems. These results are consistent with the findings in animal models, which suggest a difficult HSV-1 reactivation in CNS (differently from peripheral ganglia) [98,99]. These studies show the value of organoid systems in recapitulating some aspects of host-pathogen interaction in human herpesvirus infections.

Collectively, these studies demonstrate that human iPSC-derived cultures can reproduce host–pathogen interactions across different infections. Although 3D cultures at this stage are not a replacement of in vivo models to study the role of adaptive immunity or the systemic manifestations of infectious diseases, they represent a valuable platform, potentially enabling disease modeling and screening of new therapeutics. We expect that improvement of organoid systems, particularly by the differentiation of immune and inflammatory cells within their architecture, will expand the variety of 3D-based infectious disease models. We posit that the current limits of translating the findings from animal models into therapies due to species-specificity of host-pathogen interactions will promote iPSCs-based models in infectious disease research.

## 5. Models of Brain Cancer

Glioblastoma multiforme (GBM) is the most lethal primary brain tumor in adults. With a 5-year survival rate of less than 5% [100] median survival rates have almost not changed in the last 30 years. Chemo- and radiotherapy combined with brain surgery still remain the main therapeutic options, though associated with low clinical response and important side effects [101].

Genetically engineered mice, tumor spheroids and patient-derived xenograft models have improved our understanding of brain tumorigenesis [102]. However, limited donor availability, cancer progression away from human genetic and epigenetic signatures [103], poor clinical translatability and genetic heterogeneity of donor patients have prompted the search for additional models [104]

Such as 3D cultures. Organoid cultures reproduce complex three-dimensional features and tumor host-cell interactions that may resemble brain cancer microenvironment, not recapitulated in standard 2D conditions [105]. Tumor microenvironment greatly influences cancer cell proliferation and incorporates several cellular lineages including macrophages, endothelia, pericytes, and fibroblasts [106], not fully present in neural organoids at this stage. Nonetheless, human brain organoids have already proved their validity in investigating tumor growth in cancer chimeric models and genetic alterations that underlie the putative initial genetic events of tumorigenesis [107]. Indeed, different researches involve iPSC-, ESC- and cancer stem cell-derived brain organoids generated to model brain glioblastoma and develop high throughput drug screening platforms.

In a study by Hubert et al., neural organoids derived from different regions of patients’ GBM samples have been differentiated to recapitulate features of tumor microenvironment using a modification of Lancaster’s protocol [108]. These organoids show different grades of tumor cell infiltration consistent with the patient distinct tumor subregions of origin, maintaining functionally diverse cancer cell populations within organoids’ architecture. In addition, GBM organoids display regional heterogeneity with an outer area of rapidly dividing cells and a hypoxic core of both differentiated senescent cells and dormant glioma stem cells (GSCs). Radiation therapy on tumor organoids reveals heterogenous tumor radiosensitivity with sensitive differentiated GBM cells and radioresistant GSCs, similarly to results in vivo [108,109]. Thus, cancer stem cell-derived organoids recapitulate aspects of glioma heterogeneity and tumorigenic microenvironment in vitro.

Glioblastoma tumorigenesis has been investigated also in chimeric models exploiting CRISPR-cas9 technology combined with ESC-derived cerebral organoids [110] Particularly, glioblastoma within neural organoids can be generated by simultaneously disrupting TP53 tumor suppressor and expressing oncogenic HRasG12V in a small number of cells. These organoid-derived cancer cells, when transplanted into the hippocampi of immunodeficient mice, exhibit tumoral molecular signatures and tumorigenic properties such as rapid progression and invasiveness within murine brains, increasing rodents’ mortality. In addition, these organoid-derived tumor cells can invade blood vessels and strongly induce angiogenesis. In this case, neural chimeric models help to elucidate some early genetic events of tumorigenesis and the tumor initiation of human gliomas, normally not investigable in humans.

In another study, human neural organoids from iPSCs and ESCs co-cultured with different patient-derived GSCs lineages display variable degrees of tumor invasiveness, consistent with GSC line-specific behavior in vivo as seen in autopsy and surgical samples [105]. Moreover, tumor brain organoids present abundant necrosis, mirroring human glioma histology [111] and a rich microtube network [105], providing potential routes for tumor propagation in vitro and resembling glioma growth in vivo [112]. Considering chemio- and radiosensitivity differences between GBM organoids and 2D GSC-cultures, the organoid group shows high resistance to chemotherapy and radiation-induced genotoxicity compared with 2D cultures, as seen often in vivo, suggesting a role of 3D microenvironment in drug resistance [105] (Figure 2). Brain tumorigenesis and chemotherapeutic drug screening have been investigated also in an iPSCs-based neural organoid model of primitive neuroectodermal tumor (CNS-PNET)-like neoplasm and GBM [50]. Particularly, by combining genome editing techniques (CRISPR-cas9 and Sleeping Beauty transposon system), tumors have been generated with the introduction of genetic aberrations in a small fraction of cells within the organoid, mimicking tumor initiation in humans. Organoids overexpressing MYC show a transcriptional and histological phenotype resembling CNS-PNET; these CNS-PNET organoid-derived cells show a distinctive genetic signature compared with organoids presenting a combination of overexpressed and knockout genes relevant for GBM. Indeed, by evaluating expression levels of invasion-related genes in tumor cells from CNS-PNET and GBM organoids, the glioma group expresses more genes involved in invasiveness, correlating with the lower infiltration propensity of neuroectodermal neoplasms in contrast to high-grade gliomas in vivo [113]. To demonstrate the value of this system in chemotherapeutic drug screening, tumor organoids have been treated with epithelial growth factor receptor (EGFR)-inhibitors, which reduce tumor growth only in GBM organoids overexpressing EGFR [50].

Collectively, these studies highlight the validity of 3D cultures in recapitulating the initiating events in brain cancer tumorigenesis, elucidating some aspects of tumor host-cell interactions as well as the utility of neural organoids in drug testing.

## 6. New Frontiers in Neural Organoid Research: Human-Animal Chimeras

The development of 3D-based technologies has provided advancements in terms of maturation, cellular complexity and diversity of neural models. However, the spatial confinement of organoid-based disease models to tissue cultures has limited the study of their interactions with the immune, circulatory and endocrine system as well as the intricate molecular network present in vivo [114]. Moreover, compared to neural stem cell niches in vivo, organoids lack afferentation structures and tend to self-organize in vitro, missing both sensory signals conveyed through the host neural system and important morphogenetic cues [110]. Lately, xenotransplantation of human iPSC-derived cells and organoids into animal models have addressed these restrictions. Particularly, human cells from iPSCs have been engrafted into immunocompromised mice to generate “humanized” chimeric models. Human-rodent models based on iPSCs have proved to be valuable tools faithfully recapitulating aspects of human disease in different organs by generating liver [115], solid tumors [116], pancreas [117], lung [118], haemopoietic [119], retinal [120] and neural chimeras [107,121].

Here, we focus on the description of neural chimeras generated by in vivo transplantation of iPSC-derived neural cells and organoids into animal models and their potential use in disease modeling.

### Neural Chimeras

Engraftment of human neural tissue into animal models has been used for decades to study human diseases [122]. The development of iPSC-based technologies, in addition to the propensity of human iPSC cultures to differentiate towards neural tissue and to engraft organs, offered the opportunity for new stem cell-based therapies [39,123]. Neurons derived from iPSCs form axonal projections with functional synapsis connectivity and integrate within the host neural circuitry when xenografted into the developing mouse brain [40]. In the last few years, different studies have shown the potential use of iPSC-based therapies in neurological diseases. For instance, both human and non-human dopaminergic neurons from iPSCs engraft in rat brains, improving functional performance in a model of Parkinson disease (PD) [124]. Moreover, human iPSC-derived dopaminergic neurons autologously transplanted into primates show long-term neuronal survival in vivo [124]. This solid evidence has provided compelling preclinical basis for a currently ongoing human clinical testing with iPSC-derived dopaminergic precursors in PD patients [10]. In a chimeric model of ischemic stroke, transplanted neural stem cells from iPSCs differentiate into astrocytes and neurons, restoring part of the impaired neurological function [125].

Lately, xenotransplantation of human 3D organoids has been characterized in murine models. Organoid-like structures can self-organize within developing mouse cortex after engraftment of iPSC-derived neural stem cells grown in a three-dimensional artificial extracellular matrix [126]. These data have been further expanded by successful transplantation of neural organoids into rodent brains showing enhanced survival, multilineage differentiation and vascularization if compared with transplantation of neural precursor cells [127]. In a recent study, intracerebral xenotransplantation of human iPSC-derived brain organoids has been characterized in nonobese diabetic-severe combined immunodeficient mice [128]. Neural organoids show successful engraftment within the murine cortex demonstrating a robust vascularization from host brain. Moreover, in long-term analysis up to 9 months, transplanted organoids display progressive neuronal maturation and differentiation, the formation of long-range axon projections and a host-graft functional neural network responsive to physiological stimuli [128]. Although it’s still debatable whether neural organoids can restore specific damaged or degenerated regions, the possibility of obtaining functional neural circuits between graft and host cells provide an alternative for modeling complex neurological disorders in vivo.

An important study illustrates the use of neural chimeras to model neuropsychiatric diseases, particularly Down syndrome (DS). IPSC-derived ventral forebrain organoids generated from Down syndrome patients show an increased production of OLIG2neural precursor cells; these cells transcriptionally drive neuronal differentiation towards GABAergic-interneurons [115], whose imbalance probably play a role in cognitive symptoms in DS in humans [104]. Accordingly, chimeric rodents xenografted with human DS organoids show an overabundance of GABAergic-interneurons and impaired performance in memory tests; both phenotypes can be reversed by inhibiting OLIG2 expression [115]. In this case, the development of human-rodent chimeras helps to investigate the interplay between abnormal gene expression and human interneuron development in vivo, offering new insights into disease pathogenesis of DS and into the modulation of specific genetic targets (OLIG2) for a potential fetal therapy for Down syndrome.

Altogether, these studies highlight the feasibility of transplanting iPSC-derived cells and organoids into chimeric mice, the advantages in terms of vascularization, cell maturation and neural network development within engrafted organoids as well as the potential use of neural chimeras in disease modeling.

## 7. Conclusions

We have provided an overview on the recent advances in modelling neurological disorders with the use of iPSC derived neural organoids. Despite striking achievements, neural organoid systems still present important limitations (Table 2).

For example, the derivation of different and reproducible brain regions within the organoid is still a challenge. Multiple protocols are being developed to overcome this issue and tackle heterogeneity; some of these are based on specific neural patterning through the use of small molecules that direct the cells towards a specific fate such as midbrain, spinal cord and hippocampus [29,30,32]. Other technologies have been optimized to ensure the intake of trophic factors into the organoid core through the use of microfluidic technologies [129] 

Moreover, organoids generated with available protocols don’t grow beyond the equivalent of a early prenatal stage, which could represent a limitation for modelling diseases that onset after birth or during adulthood. Indeed, the lack of vascularization in the neural tissue hinders long-term maturation of neural organoids. In vivo transplantation of human neural organoids into adult murine brains [128] or organ buds generated by combining specific neural progenitors with mesenchymal stem cells [130] represent valuable solutions.

Brain organoids are deprived of structures that provide spatial orientation during development. The advancement of bioengineering techniques offers interesting perspectives for the production of specific scaffolds [131] able to support and guide the growing tissue and that can be selectively permeated by gradients of small molecules.

In addition, methods to maximize the variety of cell populations within the organoid have been recently published focusing on the development of microglia [132] and oligodendrocytes [133].The deficiency of inputs from the sensory system can be partially overcome by generating interconnected assembloids [134] or transplanting organoids within a host neural circuit [126]. This strategy ensures a proper amount of blood flood and synaptic inputs to the graft, which can be studied in its interaction with the host environment.

Although organoids are in principle amenable to high-throughput screenings, current methods require technical efforts and important manipulations that have hindered progress. Researchers have used organoids modelling epithelial cancers [135,136] and kidney [137] for drug discovery with high-throughput approaches, but well-established and validated methods exploiting neural organoids still lack. Recently, Qian et al. have optimized existing protocols to generate neural organoids as potential platforms for drug discovery [29]. A key advancement in organoid technology has been the use of spinning bioreactors to enable nutrient and oxygen diffusion, increasing the size and complexity of organoids [46]. Unfortunately, most of available spinning bioreactors require a consistent supply of medium over months of culturing and much incubator space, increasing the cost. These obstacles preclude scalability and compound screening in many laboratories, and they limit the possibility of testing different conditions to optimize protocols. Qian et al. have used a low-cost, miniaturized spinning bioreactor system to generate hypothalamic, midbrain and forebrain organoids by exposition to different patterning factors [29]. Forebrain organoids differentiated with this system have been exposed to ZIKV at various developmental stages. Infection of neural progenitor cells in early phases directly correlates with reduced organoid size, decreased neuronal thickness and dilated ventricles as demonstrated in previous researches using different bioreactors [29,90]. This system could serve as low cost and effective platform for drug discovery using neural organoids.

Despite these limitations, neural organoids have already provided valuable insights into neurological disorders offering a platform to investigate cellular interactions and circuit dysfunction. The possibility to genetically manipulate organoids have allowed to study the pathogenetic effect of disease causative mutations and test potential therapeutic compounds.

Altogether, these results suggest that the creation of refined and complex brain organoids will allow a deeper investigation of human neural development and pathology. Moreover, they can be a precious tool to study patients’ specific mutations and epigenetic profile with the ultimate aim to design personalized therapeutic strategies. Further researches will be needed to optimize and refine 3D system models for extensive translational application.

## Figures and Tables

**Table 1:** Selected studies investigating neurological and neuropsychiatric disorders using human iPSC-derived 3D organoids.

Disease	Organoid Type	Days of Differentiation	Phenotype and Rescue	Unique Experimental Feature	Protocol
Timothy Syndrome [41]	Ventral and dorsal forebrain, assembloids	4 weeks	GABAergic interneuron abnormalities: altered saltation frequency and shorter saltation length; phenotype rescue by pharmacological modulation of l-type calcium channels	Forebrain assembloids with labelling of specific cell type (Dlxi1/2b::eGFP)	[42]
Autism Spectrum Disorder [43]	Dorsal forebrain	6 weeks	Transcriptome dysregulation: FOXG1 upregulation; increased production of NPCs and GABAergic neurons; rescue by shRNA attenuation of FOXG1 expression	Lentiviral-mediated expression of shRNA-FOXG1	[23]
Miller-Dieker Syndrome [44]	Forebrain	4 weeks	Smaller organoids with reduced neuroepithelial loops, impaired vRG divisions, disrupted cortical niche; rescue by gene re-expression or β-catenin activation	Doxycycline-inducible overexpression of LIS1 gene	[45,46]
Autosomal recessive primary microcephaly [22]	Undirected	~3 weeks	Altered vRG morphology and orientation, smaller organoids; rescue by shRNA for CDK5RAP2	Electroporation-mediated overexpression of CDK5RAP2 and shRNA-CDK5RAP2	[22]
Hypoxic injury [47]	Forebrain	~11 weeks	Disruption of intermediate progenitors in SVZ; impaired UPR pathway activation and premature neuronal differentiation; rescue by stress response inhibitor (ISRIB)	Gas control chamber and needle-type fiber-optic microsensor to reproduce and monitor low oxygen exposure	[42]
Zika virus infection (ZIKV) [29]	Forebrain	~3 months	Smaller organoids with reduced thickness and increased ventricular lumen, ZIKV-induced cell apoptosis	ZIKV strains: MR766 and FSS13025 (99% amino acid sequence homology to Brazilian ZIKV)	[29]
Cytomegalovirus infection (CMV) [48]	Undirected	8 weeks	Reduced cell proliferation, necrosis, vacuolar and cystic degeneration; impaired cortical lamination	Organoid differentiation from CMV-infected hiPSCs	[46]
Creutzfeld-jakob disease (CJD) [49]	Undirected	5 months	Slow metabolism, protease-resistant PrP deposition, acquired prion seeding activity, increased astrocyte activation	Organoids inoculation with human brain homogenates from sporadic CJD subtypes	[46]
Brain tumors [50]	Forebrain	~4 months	Glioma-like with poor glial differentiation, high cell proliferation, disorganized architecture and downregulation of PI3K-AKT, RAS pathways; CNS-PTEN-like with WNT, TGFβ, and TP53 upregulation; tumor invasiveness upon in vivo transplantation; partial rescue in glioma by EGFR-inhibitors	Electroporation-mediated plasmid nucleofection with overexpression of MYC/inhibition of tumor suppressor genes	[22]

**Table 2:** Pros and cons of neural organoids and potential solutions/current approaches to the major limitations of the organoid model.

PROS	CONS	APPROACH
3D multicellular architecture with complex cell compartmentalization	Lack of reproducibility	- Microfluidic technologies modulating local stimuli to cellular microenvironment - Patterned organoids
Patterning into different brain-like subregions	Cost	- Miniaturized spinning bioreactors with reduced incubator space and medium supply needed
Rough organization into cortical layers	Lack of output and input systems	- In vivo transplantation - Development of assembloids
Long term culturing	Lack of vascular bed	- Combined progenitors (mesenchymal and neural stem cells) - In vivo transplantation into animal models
Generation of patients’specific disease-relevant cell types	Spatial orientation	- Bioengineered scaffolds
Generation of spontaneously active neural networks	Long term maturation	- In vivo transplantation - Optimization of culture conditions and culture media
